# Prolonged Delirium in a Young Migrant with Graves’ Disease: The Role of Thyroid Assessment in Neuropsychiatric Presentations

**DOI:** 10.1192/j.eurpsy.2026.11804

**Published:** 2026-07-31

**Authors:** P. M. Martins, A. Samouco

**Affiliations:** Department of Psychiatry, ULS Tâmega e Sousa, Porto, Portugal

## Abstract

**Introduction:**

Thyroid dysfunction can manifest through diverse neuropsychiatric symptoms, often resembling primary psychiatric disorders. Early identification of endocrine causes is essential to avoid misdiagnosis and ensure appropriate treatment.

**Objectives:**

To highlight the importance of thyroid evaluation in acute neuropsychiatric presentations.

**Methods:**

Case report.

**Results:**

A 23-year-old Venezuelan man, recently emigrated to Portugal and with no psychiatric history, presented with a three-day history of psychomotor agitation, disinhibition, insomnia, disorganized thinking, and bizarre behavior. Examination revealed alertness with disorientation, distractibility, unkempt appearance, and poor cooperation. Initial laboratory testing showed undetectable TSH, elevated free T3 and T4, and positive thyroid antibodies (TRAb 17.87 IU/L; TgAb 17.5 IU/mL; TPOAb 313.1 IU/mL). Neuroimaging findings were unremarkable, and toxicology screening was negative. Based on these results, a diagnosis of Graves’ disease was established, and appropriate treatment was initiated. Despite achieving euthyroidism, agitation, confusion, and distractibility persisted. Further investigations, including electroencephalography, brain magnetic resonance imaging, and cerebrospinal fluid analysis, yielded unremarkable findings. No infectious, metabolic, or structural cause was found. Hashimoto’s encephalopathy was suspected. Corticosteroid therapy was initiated; however, it led to clinical worsening, with increased agitation and affective lability. Gradual tapering and discontinuation of corticosteroids resulted in behavioural improvement. Transient use of antipsychotic medication was required for agitation but was later withdrawn. At discharge, the patient showed partial improvement, with residual cognitive rigidity and continued need for supervision in daily activities.

**Conclusions:**

This case illustrates the intricate relationship between thyroid dysfunction and neuropsychiatric manifestations. Thyrotoxicosis may precipitate cognitive and behavioural disturbances that closely mimic primary psychiatric disorders, while autoimmune thyroid disease can also be associated with encephalopathic processes of immune origin. The persistence of neuropsychiatric symptoms despite normalization of thyroid function emphasizes that thyroid-related brain dysfunction may have a multifactorial and prolonged course, extending beyond metabolic imbalance alone. Prompt recognition of reversible thyroid-related causes is crucial for timely, multidisciplinary management and better outcomes.

**Disclosure of Interest:**

None Declared

